# Coronary artery disease in a patient with Addison’s disease: a case report and literature review

**DOI:** 10.1186/s12872-023-03079-0

**Published:** 2023-01-29

**Authors:** Ruohan Zhao, Suxin Luo, Shuzhen Wang, Yi Wen, Feng Xiong

**Affiliations:** 1 Department of Cardiology, Cardiovascular Institute of Chengdu Third People’s Hospital/The Affiliated Hospital of Southwest Jiaotong University, Chengdu, 610031 China; 2 Department of Cardiology, The First Affiliated Hospital of Chongqing Medical University, Chongqing, 400016 China; 3 Health Management Centre, University-Town Hospital of Chongqing Medical University, Chongqing, 401331 China

**Keywords:** Addison’s disease, Adrenal tuberculosis, Unstable angina, Coronary artery disease, Case report

## Abstract

**Background:**

Addison’s disease which is due to dysfunction of the adrenal gland, with abnormal secretion of glucocorticoids and mineralocorticoids, is rare. By inducing inflammation and disorders of water and electrolyte metabolism, Addison’s disease may accelerate progression of co-existed cardiovascular diseases. Addison’s disease combined with cardiovascular disease is infrequent, only 10 cases in the literature.

**Case presentation:**

We reported a 51-year-old male patient with unstable angina pectoris and hypotension. Changes on coronary angiography within 2 years suggested rapid progression of coronary artery disease in a patient with low cardiovascular risk. An additional clue of skin hyperpigmentation, fatigue and further examination confirmed the diagnosis of Addison’s disease caused by adrenal tuberculosis. After hormone replacement treatment, the frequency and severity of the angina pectoris were alleviated significantly, as were hypotension, hyperpigmentation and fatigue.

**Conclusions:**

The combination of Addison’s disease and coronary artery disease in one patient is rare. Addison’s disease can induce inflammation and disorders of water and electrolyte metabolism, which may further accelerate the course of coronary artery disease. Meanwhile, the hypotension in Addison’s disease may affect the coronary blood flow, which may result in an increased susceptibility to unstable angina in the presence of coronary stenosis. So, we should analyze comprehensively if the coronary artery disease progress rapidly.

## Background

Primary adrenal insufficiency (PAI), known as Addison’s disease, was first reported by Thomas Addison in 1855 [1]. The prevalence is about 10–15 cases per 100,000 [2]. The main cause of Addison’s disease in developed countries is autoimmune disease, accounting for 70–90% [3]; But in China, adrenal tuberculosis accounts for 58.1% [4]. With the prevalence of unhealthy lifestyles in China, the incidence and mortality of coronary artery disease (CAD) remain high [5]. Hypertension, dyslipidemia, and inflammation are closely related to the process of atherosclerosis. Adrenal insufficiency leads to inadequate secretion of glucocorticoids and mineralocorticoids, which further result in disorders of water, electrolyte, glucose and lipid metabolism and inflammation [2]. The disorder of hormone could accelerate the course of CAD. The co-existing incidence of CAD and Addison’s disease is 6.9–10 cases per 1000,000 [6], which is rare. This article introduces a case of CAD combined with Addison’s disease, with manifestations of chest pain and hypotension. In addition, we provided review of related literatures.

## Case presentation

**Fig. 1:**
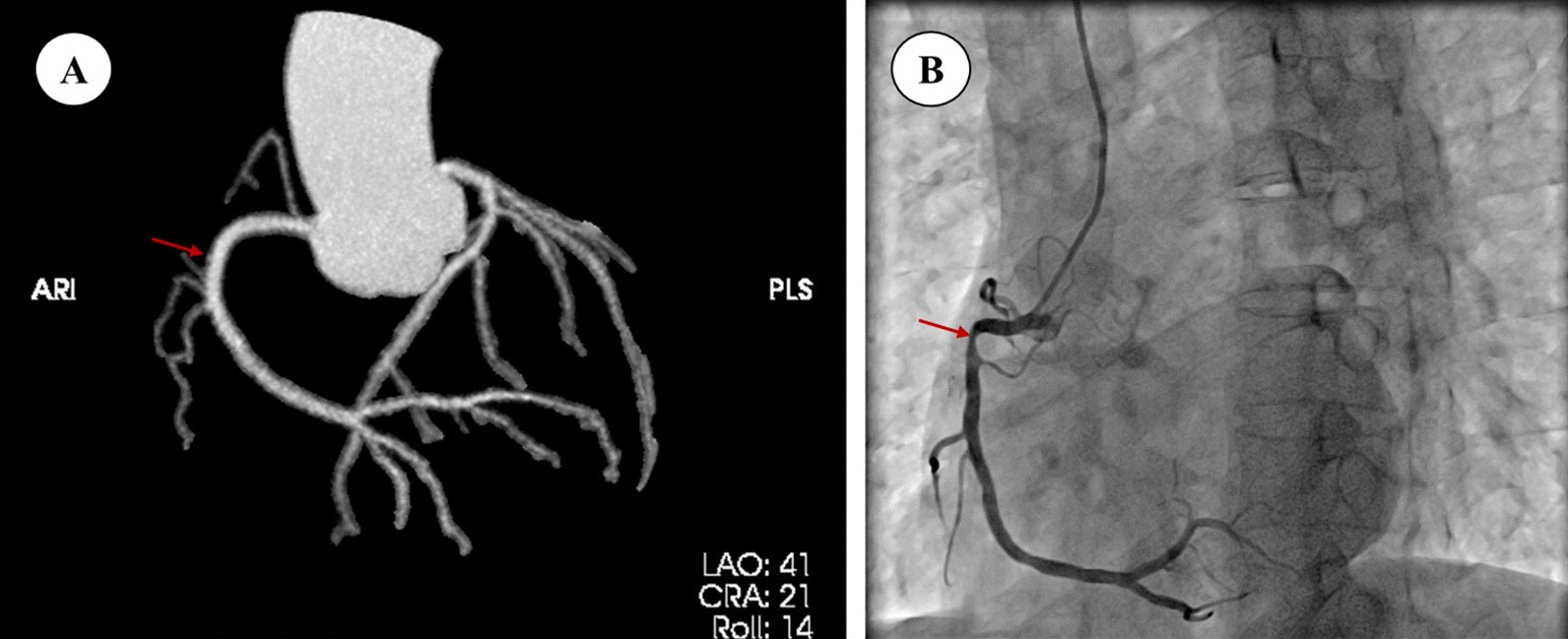
**A** two years ago, coronary artery CTA showed the right coronary was almost normal. **B** two years later, coronary angiography showed 80% stenosis (red arrow) of the proximal right coronary artery

The patient was a 51-year-old male admitted to the hospital for recurrent chest pain for 2+ years, which aggravated with fatigue for 3+ months. More than 2 years earlier, the patient noticed chest pain while climbing the mountain (CCS Grade I), which radiated to the back. The coronary computed tomography angiography (CCTA) indicated the coronary artery was almost intact (Fig. 1A). He was treated with simvastatin. Three months before the hospitalization, the patient experienced chest pain at rest (CCS Grade II), with increased duration and frequency. The angina pectoris could be relieved by nitroglycerin. Coronary angiography showed 80% stenosis of the proximal right coronary artery (Fig. 1B), 40% stenosis of the proximal circumflex artery, and 30% stenosis in the middle of the anterior descending branch. Then local hospital prescribed him aspirin + clopidogrel + perindopril + metoprolol, nitroglycerin at necessary.

**Fig. 2:**
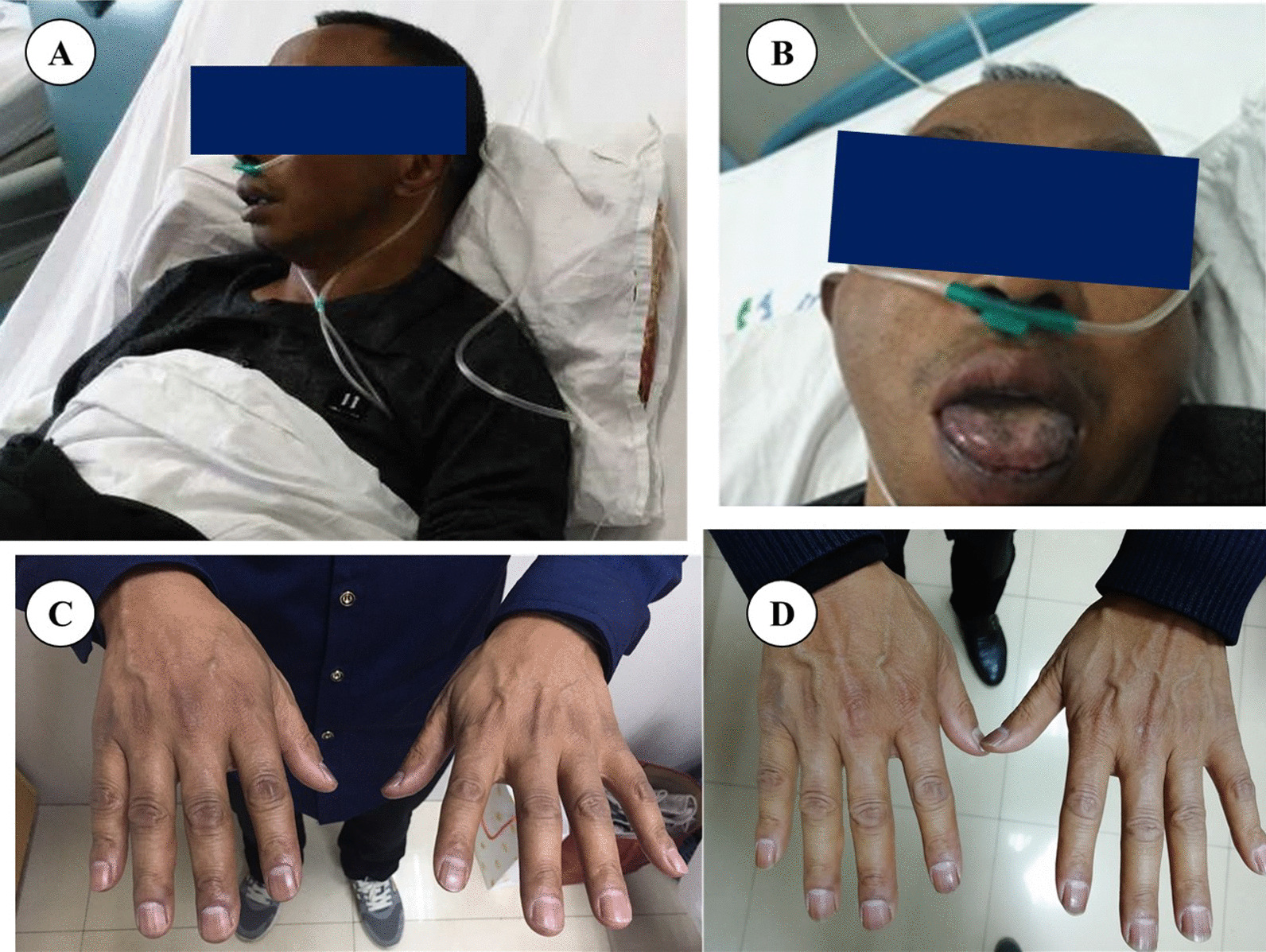
Skin and mucosal pigmentation changes in patients with Addison’s disease. **A** Diffuse dark-brown changes in facial skin; **B** Dark-brown spots of different sizes visible on the lips and tongue; **C** Diffuse dark-brown changes on the back of the hands; **D** After 2 months of treatment, the pigmentation on the back of the handss was partially reversed

The patient had smoked roughly 20 cigarettes per day for 32 years, smoking cessation for 2 years, and had no history of hypertension or diabetes. He didn’t have a cardiovascular family history. Upon physical examination, the patient presented a hypotension of 72/56 mmHg. The patient’s bare skin, lips, oral mucosa, areola, and palm lines were visibly pigmented (Fig. 2A–C). No other physical examination was remarkable. Upon further inquiry into the patient’s medical history, the marked hyperpigmentation of aforementioned areas developed 3 months ago, accompanied by decreased appetite and weakness of the limbs. A chest CT scan performed 2 months earlier revealed old pulmonary tuberculosis.

**Fig. 3:**
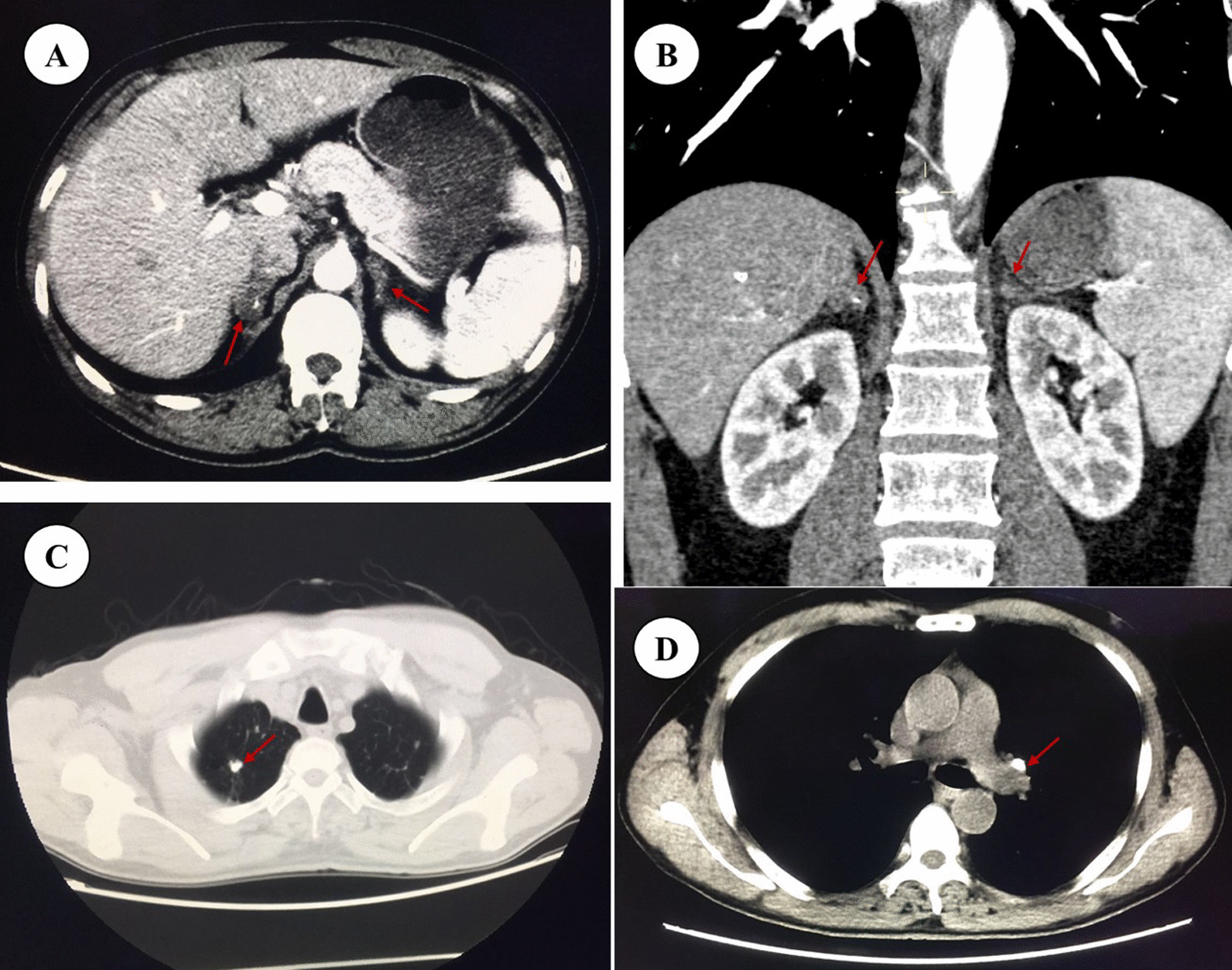
Enhanced CT findings of the chest and abdomen in Addison patients. **A**, **B** Visible irregular thickening of the bilateral adrenal glands with a few punctate calcifications (red arrows); **C** Multiple small nodules, cords, and calcifications in the upper lobes of the lungs (red arrow); **D** Multiple calcifications in the left hilar and mediastinum lymph nodes (red arrow)

**Fig. 4:**
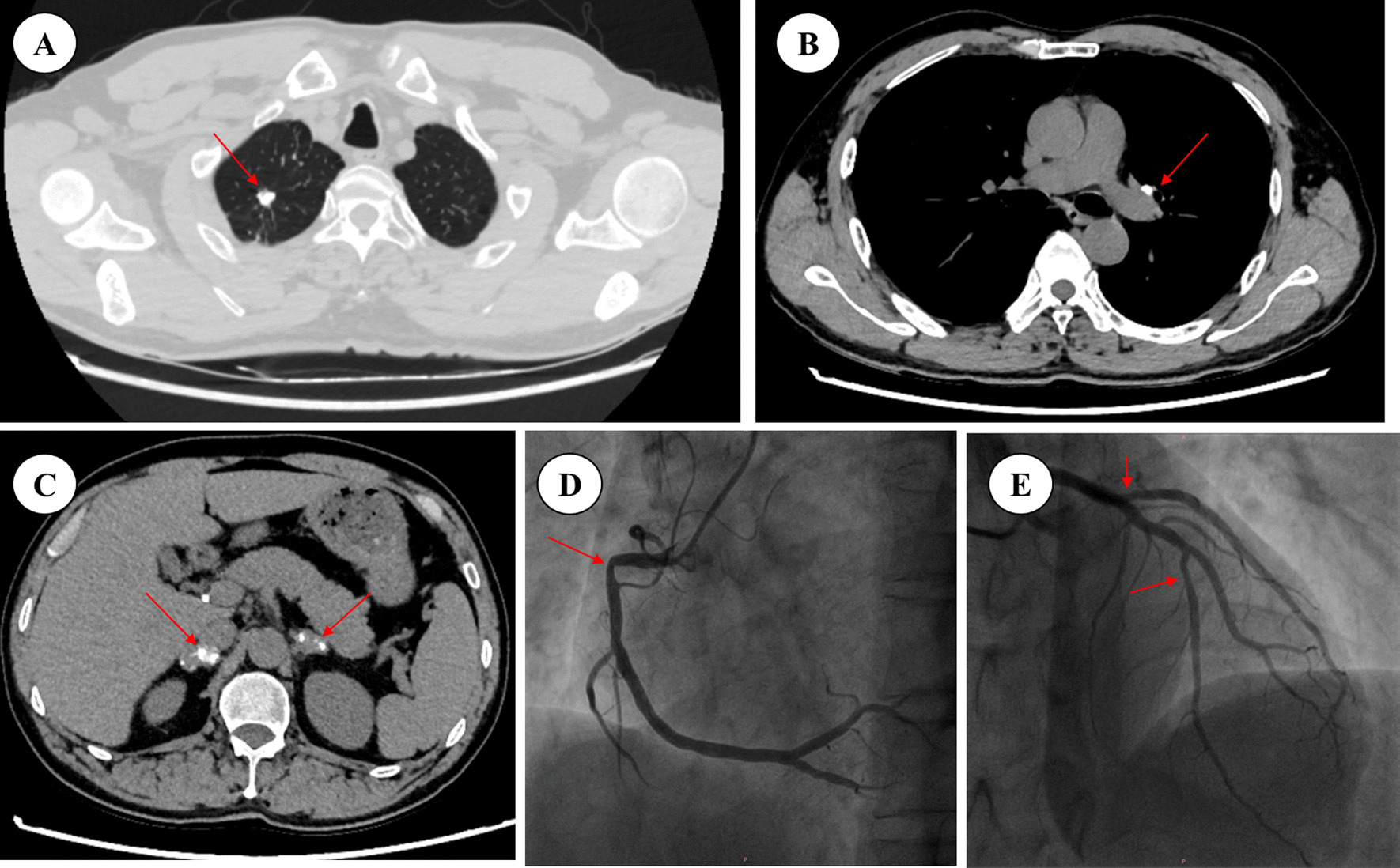
Patient’s follow-up images. **A**, **B** Pulmonary tuberculosis and adrenal tuberculosis are stable. (red arrow showed the calcification in the upper lobes of right lungs and lymph node); **C** Calcification of adrenal tuberculosis increased (red arrow). **D** A stenosis of 60% in the proximal segment of right coronary artery (red arrow); **E** A stenosis of 40–50% in the distal segment of anterior descending branch, a stenosis of 20–30% in the middle segment of circumflex artery (red arrow)

**Fig. 5:**
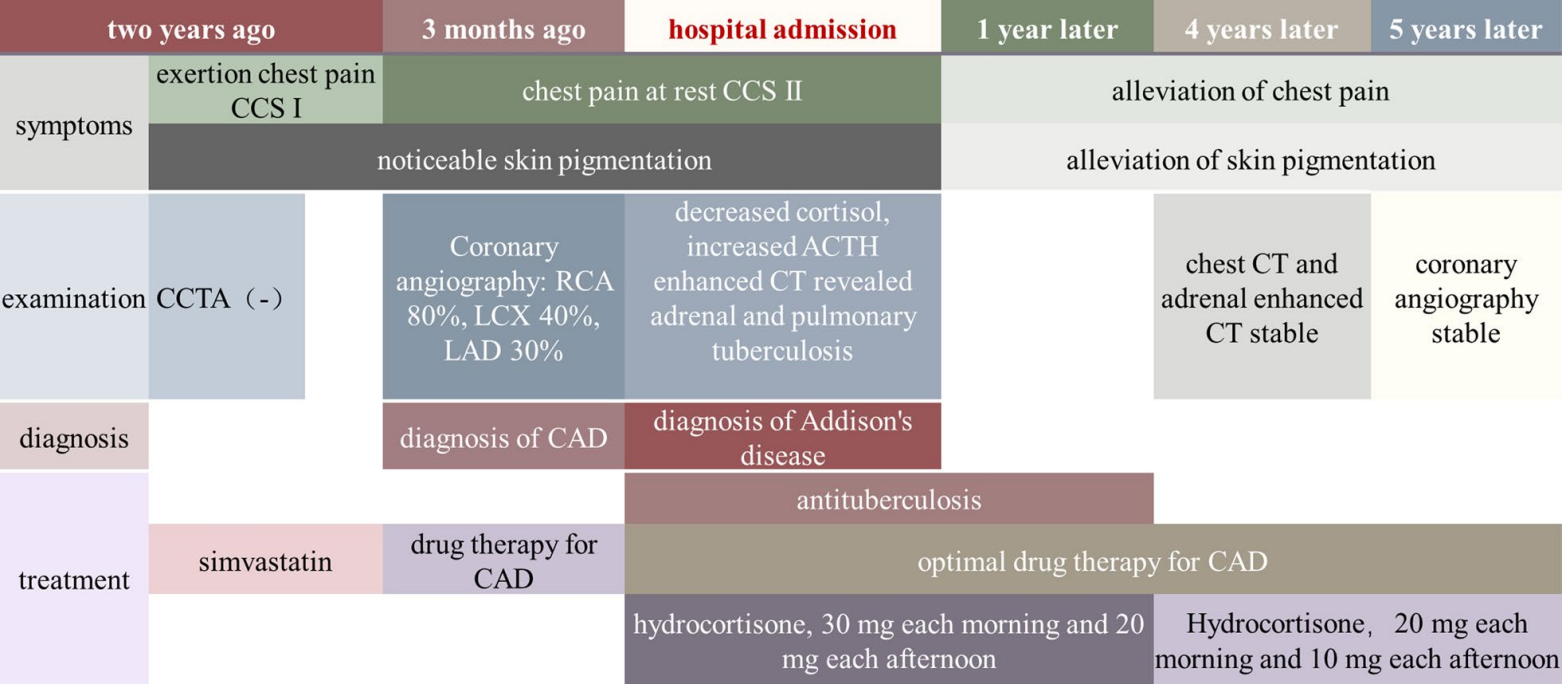
Time line table of the patient

Admission examination results were as follows: fasting blood-glucose 4.6–5.5 mmol/L (reference 3.9–6.1 mmol/L); HbA1c 5.7%; TC 2.44 mmol/L (reference 2.80–5.20 mmol/L); TG 1.31 mmol/L (reference 0.35–1.70 mmol/L); HDL-c 0.79 mmol/L (reference 1.29–1.55 mmol/L); LDL-c 1.45 mmol/L (reference 0–3.37 mmol/L); and Na 134 mmol/L (reference 135–145 mmol/L). The ambulatory blood pressure revealed a slightly low diastolic blood pressure, whose average was 91/58 mmHg, and floor level was at 70/52 mmHg. The ambulatory electrocardiogram indicated ST-T depression. Color Doppler ultrasound of the carotid arteries suggested the formation of bilateral carotid plaques. There were no obvious abnormalities in the echocardiography, blood routine findings, blood coagulation, anti-nuclear antibodies, or thyroid function. According to the clinical manifestations of the patient’s skin pigmentation, loss of appetite, fatigue, hypotension, and hyponatremia, adrenal disease was suspected. The examination found the level of cortisol significantly reduced while ACTH increased. The 24 h ACTH and cortisol circadian rhythms further indicated the abnormality of the circadian rhythm (Table 1). Enhanced CT of the abdomen showed visible uneven irregular thickening of the bilateral adrenal glands with a few punctate calcifications (Fig. 3A, B). Enhanced CT of the chest showed multiple small nodules, cords, and calcifications in the upper lobes of both lungs, mainly proliferation and calcification; the left hilar and mediastinum had multiple lymph node calcifications (Fig. 3C, D). Pulmonary and adrenal tuberculosis was considered. Tuberculosis antibody was 2+ and T-SPOT was positive. Thus, he was diagnosed with primary adrenal hypofunction caused by adrenal tuberculosis combined with CAD. Since we could not get the pathological support of adrenal tuberculosis activity status, anti-tuberculosis treatment and hormone replacement therapy were initiated. The anti-tuberculosis therapy is rifampicin 0.45 g qd + isoniazide 0.3 g qd + pyrazinamide 1.5 g qd + ethambutol 0.75 g qd for 12 months [7]. Hormone replacement therapy is hydrocortisone 30 mg each morning and 20 mg each afternoon when the anti-tuberculosis therapy persisted, while 20 mg each morning and 10 mg each afternoon after the anti-tuberculosis therapy [7]. As for the CAD, we didn’t perform PCI to right coronary artery stenosis. There were two main reasons. Firstly, the blood pressure, especially the diastolic pressure, is of low level. There is a great possibility that blood pressure will recover when adrenal function improves. After hormone replacement therapy, blood pressure and myocardial perfusion would improve, so angina pectoris would recover. Secondly, we believed that Addison disease may contribute to the rapid progression of the coronary artery stenosis as well as the aggravation of chest pain. As the control of Addison disease, the progress of the coronary plaque will be stabilized. So, an optimal drug therapy for CAD, which consist of aspirin + perindopril + metoprolol + simvastatin, was retained. After the treatment, the patient’s skin pigmentation (Fig. 2D), loss of appetite, fatigue, especially the frequency and severity of angina pectoris were ameliorated. The patient’s blood pressure fluctuated between 111–129/62 and 80 mmHg. Until the last follow-up, the patient’s chest CT (27 April, 2021) (Fig. 4A, B), adrenal enhanced CT (27 April, 2021) (Fig. 4C) and coronary angiography (27 July, 2022) (Fig. 4D, E) remained stable. The coronary angiography revealed a stenosis of 60% in the proximal segment of right coronary artery, a stenosis of 40–50% in the distal segment of anterior descending branch, a stenosis of 20–30% in the middle segment of circumflex artery. The coronary lesion did not evolve, so we persisted the medical treatment as well. The time line table of the patient’s medical process is as follows (Fig. 5).

**Table 1:** 24 h ACTH and cortisol circadian rhythm

Time	00:00 a.m	8:00 a.m	4:00 p.m
Cortisol	72.59 nmol/L	73.19 nmol/L	57.87 nmol/L
Reference	12.8–82.5 nmol/L	124.2–662.4 nmol/L	49.68–179.4 nmol/L
ACTH	352.1 pg/mL	1478 pg/mL	856.8 pg/mL
Reference	7.2–63.3 pg/mL	7.2–63.3 pg/mL	7.2–63.3 pg/mL

## Discussion and conclusion

This case is unique because Addison’s disease sped up the progression of CAD and was engaged in the cause of chest pain. 2 more years before the admission, the coronary CTA indicated only mild coronary artery stenosis, and then lipid-lowering and antiplatelet therapy were initiated. Within 2 years, the mild stenosis of coronary artery progressed to 80% stenosis of proximal right coronary, 40% stenosis of the proximal circumflex branch, 30% stenosis of the middle anterior descending branch. At first, the angina pectoris could only be induced by labor, which subsquently progressed into unstable angina. Considering that the patient had received CAD drug therapy, with normal cholesterol level and glucose metabolism, the only suspected risk factor was history of smoke; a study by Ding et al. comprehensively compared the long-term association of cigarette smoking and its cessation with the incidence of CAD. Besides a dose–response relationship of pack-years of smoking and CAD, risk of CAD elevated significantly up to 20 years after smoking cessation [8]. However, the coronary stenosis progression was significantly accelerated after smoking cessation, especially after the anti-atherosclerosis therapy. Angina pectoris worsened with the aggravation of Addison’s disease, and improved after hormone replacement therapy, so we believed there may be a connection between CAD and Addison’s disease.

Therefore, we searched the literatures from 2006 to the present. A total of 11 cases of Addison’s disease with heart disease were found (current case included) (Table 2). Female patients accounted for a high proportion (63.6%). Adrenal tuberculosis caused adrenal insufficiency was found in 3 cases (27.3%). The final cardiac diagnoses were 2 cases (18.2%) of angina pectoris, 2 cases of myocardial stunning or hibernation (18.2%), 4 cases of takotsubo heart disease (36.4%), 1 case of ST-segment elevation myocardial infarction and stent implantation (9.1%), and 1 case of coronary artery spasm (9.1%). Nine cases showed changes in the electrocardiogram (81.8%). Two cases (18.2%) were positive for coronary angiography. Seven cases (63.6%) presented segmental dyskinesia of the ventricular wall in echocardiography. All cases showed Addison’s disease, which may affect the course of cardiovascular disease. The disparity of our case is that: Firstly, from the coronary imaging evidence of the recent 2 years, we can see intuitively the rapid progression of CAD with the combination of Addison’s disease. Secondly, the patient’s chest pain was alleviated by simply hormone replacement therapy rather than a stent implantation.

**Table 2:** Characteristics of patients with Addison’s disease complicated by cardiovascular disease

Resource	Gender	Age (year)	Etiology of API	ACTH	Cortisol	Symptom (s)	ECG	Myocardial enzyme	Angiography	Echocardiography	Cardiac diagnosis
Current case	M	51	Adrenal tuberculosis	↑	↓	Chest pain at rest	(–)	(–)	Proximal right coronary artery 80%, proximal circumflex artery 40%, middle of the anterior descending branch 30%	(–)	Angina pectoris
Iga. et al. [18]	F	74	Adrenal crisis induced by operation	↓	↓	Fatigue, loss of appetite	Deep negative T wave	(–)	(–)	Akinesis of antero-septal and LV apical area	Myocardial hibernation or stunning
	F	64	Adrenal crisis induced by hypoglycemia	↓	↓	Loss of consciousness	ST elevation in the left precordial leads	(–)	(–)	Aneurysm in the antero-septal and apical region of LV	Myocardial hibernation or stunning
Ozcan. et al. [19]	F	39	NK	↑	↓	Nausea, vomiting, weakness, and hyperpigmentation	ST depression and inverted T wave on inferior and V4–V6	(–)	(–)	(–)	ECG changes caused by adrenal crisis
Akpa. et al. [20]	M	48	Adrenal tuberculosis	↑	↓	Chest pain, nausea, vomiting, and difficulty breathing	Low voltage, ST elevation of 1.5 mm in all chest leads	Slight elevation of LDH, normal CPK	Not complete	Not complete	Angina pectoris
Punnam. et al. [21]	F	71	NK	–	–	Weakness, fatigue, and lightheadedness	ST elevation in V2–V6	↑	(–)	Dyskinetic apical and inferior walls, EF25-30%	Takotsubo cardiomyopathy induced by Adrenal crisis
Barcin. et al. [22]	F	41	NK	–	–	Chest pain	(–)	↑	(–)	LV apical akinesis, EF 44%	Takotsubo cardiomyopathy
Singh. et al. [23]	M	48	Pituitary adenoma	↓	↓	Difficulty breathing, vomiting	ST elevation and T wave inverted in the lateral leads	↑	(–)	RWMAs involving left anterior descending territory, low EF	Takotsubo cardiomyopathy
Campean. et al. [24]	F	41	II -APS	↓	↓	Shortness of breath	Tachycardia, inverted T wave in V5, V6, prolongation of cQT	↑	Not complete	Dyskinetic apical lateral and inferior walls of LV, EF 30%	Takotsubo cardiomyopathy
Maranduca. et al. [17]	F	71	Possible adrenal tuberculosis	↑	↓	Chest pain	q wave in I, aVL, V5, V6, ST elevation in V2–V5	(–)	Not complete	LV apical and inferior wall akinesis EF 50%	STEMI
Otsuka. et al. [25]	M	60	Adrenalectomy	↑	↓	Palpitation, fatigue, chest pain	ST elevation, ventricular tachycardia	(–)	No significant visible stenosis, diffuse spasm of LAD after ergometrine administration	(–)	Coronary spasms

Over doses of glucocorticoids increase the risk of cardiovascular disease, but little is known about the relationship between glucocorticoid insufficiency and cardiovascular disease. The reasons for the progression of CAD due to Addison’s disease may be as following: (1) Esposito et al. show that patients with adrenal insufficiency often have low blood volume and low blood pressure [9, 10]. When combined with coronary artery stenosis, insufficient coronary blood supply would further aggravates myocardial ischemia; (2) Cortisol insufficiency is accompanied by increased levels of inflammatory cytokine such as TNF-α, IL-1, and IL-6 [11], which are closely related to the occurrence of cardiovascular events [11, 12]; (3) Cortisol insufficiency is also related to disorders of glucose and lipid metabolism. Studies have shown that the levels of TG and LDL-c in the Addison disease group are higher than those in the control group, while HDL-c is lower than that in the control group. Disorders of blood lipid metabolism are also risk factors of cardiovascular disease [13]. (4) ACTH promotes osteochondrogenic mesenchymal cell differentiation, which may contribute to the pathologic progression of calcified atherosclerosis [14]. Moreover, ACTH can also promote the aggregation of platelets, leading to the formation of acute arterial thrombosis [15]. Overall, adrenal insufficiency can impair cardiovascular system in terms of low blood volume, low blood pressure, increased inflammatory factors, dyslipidemia, coronary calcification, arterial thrombosis, etc.

The treatment in this case is also worth discussing. Firstly, as for the treatment of Addison’s disease, regular and comprehensive anti-tuberculosis therapy and hormone replacement therapy are effective methods for the treatment of adrenal tuberculosis. Once adrenal tuberculosis with Addison's disease is diagnosed, lifelong glucocorticoid replacement therapy, usually by hydrocortisone or prednisone, should be initiated immediately and maintained in the future. The main objective is to alleviate symptoms and to keep serum ACTH at a normal range. The appropriate dose of glucocorticoid should be determined according to height, weight, gender, age, physical labor intensity. Mineralocorticoids can be added when there is hypotension, hyperkalemia, and insufficient aldosterone secretion. Under stress condition, such as fever, surgery, and trauma, the dose of glucocorticoids should be increased by 2–3 times [16]. Secondly, in case where Addison’s disease was caused by adrenal tuberculosis, it is important to determine whether there is an active tuberculosis foci. Given that glucocorticoid replacement therapy may activate old tuberculosis or induce metastasis of tuberculosis foci, anti-tuberculous therapy should be routinely given for about half a year after the initial diagnosis of an inactive tuberculosis patient and given for 6 to 18 months in the active status [7]. The combination of hormone replacement therapy and anti-tuberculosis therapy presents challenges because rifampicin is a strong inducer of the cytochrome P450 system which is involved in the metabolism of adrenocortical hormones. This interaction can lead to insufficient concentration of hydrocortisone and even adrenal crisis. The Japan Endocrine Society recommends that the dose of hydrocortisone should be increased to 2–3 times the original when treating with tuberculosis, but there is no specific drug recommendation [16]. Thirdly, as mentioned above, Addison's disease may affect the progression of cardiovascular diseases through low blood pressure, increased inflammatory factors etc. pathways. Therefore, it is necessary to check whether the patient has Addison’s disease or other systemic diseases when the patient suddenly has an aggravation of cardiovascular disease. In our literature review, only 1 out of 11 patients with Addison's disease who had cardiovascular symptoms underwent stent implantation (due to myocardial infarction) [17], while the rest were able to control their cardiovascular symptoms with hormone replacement and optimized CAD medication.

In summary, comprehensive analysis is important during the differential diagnosis of chest pain. Cardiovascular considerations alone may lead to missed diagnosis of systemic disease. Although Addison’s disease caused by adrenal tuberculosis is rare, its clinical manifestations are specific. Hyperpigmentation, unexplained hypotension, unexplained hyponatremia, and hypokalemia are all crucial clues to diagnose Addison’s disease.

## Data Availability

The data analyzed are available from the corresponding author on reasonable request. A copy of the consent form is available for the Editor to review upon request.

## Abbreviations

PAI: Primary adrenal insufficiency
CCTA: Coronary computed tomography angiography
CAD: Coronary artery disease
